# No Evidence of Significant Levels of Toxigenic V. cholerae O1 in the Haitian Aquatic Environment During the 2012 Rainy Season

**DOI:** 10.1371/currents.outbreaks.7735b392bdcb749baf5812d2096d331e

**Published:** 2013-09-13

**Authors:** Sandrine Baron, Jean Lesne, Sandra Moore, Emmanuel Rossignol, Stanislas Rebaudet, Pierre Gazin, Robert Barrais, Roc Magloire, Jacques Boncy, Renaud Piarroux

**Affiliations:** ANSES (Agence nationale de sécurité sanitaire de l’alimentation, de l’environnement et du travail), Ploufragan-Plouzané Laboratory, Ploufragan and Université Européenne de Bretagne, Rennes, France; ANSES, Maisons-Alfort, France; Aix-Marseille University, UMD 3, Marseilles, France; Ministère de la Santé Publique et de la Population, Port-au-Prince, Haiti; Aix-Marseille University, UMD 3, Marseilles, France; Aix-Marseille University, UMR 912 SESSTIM (AMU, INSERM, IRD), Marseille, France; Ministère de la Santé Publique et de la Population, Port-au-Prince, Haiti; Ministère de la Santé Publique et de la Population, Port-au-Prince, Haiti; Ministère de la Santé Publique et de la Population, Port-au-Prince, Haiti; Aix-Marseille University, UMD 3, Marseilles, France

## Abstract

Background: On October 21, 2010, Haiti was struck by a cholera epidemic for the first time in over a century. Epidemiological and molecular genetic data have clearly demonstrated that the bacterium was imported. Nevertheless, the persistence of the epidemic for more than two years, the high incidence rates in some coastal areas and the seasonal exacerbations of the epidemic during the rainy seasons have prompted us to examine the levels of toxigenic Vibrio cholerae in the Haitian aquatic environment. Methods: In July 2012, during the warm and rainy season, 36 aquatic stations were sampled to search for toxigenic V. cholerae. These stations included fresh, brackish and saline surface waters as well as waste water; the sampling sites were located in both rural and urban areas (around Port-au-Prince and Gonaïves) located in the West and Artibonite Departments. V. cholerae bacteria were detected in enrichment cultures of water samples (sample volumes included 1 L, 100 mL, 10 mL, 1 mL, 0.1 mL, 0.01 mL and 0.001 mL depending on the context). Detection methods included both culture on selective agar (for strain isolation) and PCR assays targeting the genes ompW (V. cholerae species), O1-rfb and O139-rfb (O1 and O139 V. cholerae serogroups, respectively), and the cholera toxin gene ctxA, which is present exclusively in toxigenic cholera strains. Results: A total of 411 culturable V. cholerae isolates from 29 stations were obtained via selective culture; however, only one of these isolates displayed a late positive reaction with polyvalent anti-O1 serum. Positive V. cholerae PCR results were obtained from each of the 32 tested stations (a total of 77 enrichments out of 107 yielded a positive result); only one sample yielded a positive V. cholerae O1 PCR result. The cholera toxin gene ctxA was never detected via PCR with either primer pair, which includes samples derived from the two stations yielding positive O1 culture or positive O1 PCR results. Therefore, we could not demonstrate the presence of toxigenic V. cholerae O1 among the 36 stations sampled. This suggests that all water samples analyzed contained less than 10 toxigenic V. cholerae O1 bacteria per liter, a level 1000-fold below the dose that has been shown to provoke cholera in healthy adults. Conclusions: Currently, there is no evidence of a significant level of contamination of the aquatic environment in Haiti by the imported toxigenic V. cholerae O1 strain. The reemergence of cholera outbreaks in Haiti during rainy seasons is therefore more likely due to persisting outbreaks insufficiently tackled during the dry periods rather than the commonly suspected aquatic reservoir of toxigenic bacteria.

## Introduction

On October 21, 2010, Haiti was struck by cholera for the first time in over a century 1. The Haitian epidemic represents the largest national cholera epidemic of the seventh pandemic with 604,634 cases and 7,436 deaths reported from October 2010 to October 2012 2.

Epidemiological data has demonstrated an exact spatiotemporal correlation between the first reported cholera cases in Meille, a small village 2 km south of Mirebalais, and the arrival of UN Nepalese peacekeepers in Haiti 3. According to Frerichs et al. (2012), the Nepalese soldiers were exposed to a cholera epidemic in Nepal in late September just before embarking for Haiti, where they were primarily stationed in a camp near Mirebalais, situated on the banks of the Meille River 4. The initial cases were biologically confirmed as *Vibrio cholerae* O1, serotype Ogawa, biotype El Tor 5. Genetic analysis has demonstrated that the Haitian cholera isolates were almost identical to isolates collected in Nepal a few weeks prior, which displayed only one- or two-base pair differences throughout the entire genome, thereby strongly suggesting that the Haitian cholera strains were very recently imported from Nepal 6.

During the first 2 years of the epidemic, cholera was disseminated throughout almost every region of Haiti, including the most remote rural areas. Meanwhile, outbreaks seemed to be aggravated by major climatic events, such as Hurricane Tomas in November 2010 and the hot and rainy seasons of 2011 and 2012. Moreover, it appears that cholera particularly affected certain coastal areas, such as the Artibonite Delta following the floods provoked by Hurricane Tomas and the low altitude wards of Port-au-Prince during the 2011 rainy season. In contrast, a major reduction in the number of cases was observed during the dry seasons 2 ^,^ 7, when cholera transmission retracts in a few rural locations and urban quarters 8. The persistence of cholera in the country for more than 2 years associated with a seasonal exacerbation of the epidemic during the rainy seasons, especially in coastal areas, has prompted us to examine the level of toxigenic *V. cholerae* contamination in the Haitian aquatic environment.

Indeed, in the Bay of Bengal, it has been shown that the environment can play a role in the durable establishment of cholera. Studies in the 1980s and 1990s have demonstrated that *V. cholerae* species can grow in various aquatic ecosystems, such as fresh waters, brackish waters and estuaries. In such environments, *V. cholerae* species associate with phytoplankton and zooplankton in a pH- and salinity-dependent manner 9. Increases in water temperature and subsequent plankton blooms have been shown to correlate with the fluctuation in cholera cases in the Bay of Bengal 10 ^,^ 11.

To investigate whether the aquatic environment presents a major risk of cholera transmission to local populations, we conducted a microbial assessment of toxigenic *V. cholerae* O1 levels on a panel of water samples isolated from several areas in the West and Artibonite departments. These areas were selected because they were heavily affected by cholera during either the floods that followed hurricane Tomas in 2010 (Artibonite Department) or the 2011 rainy season (Port-au-Prince and Gonaïves areas). Most of the sample sites were coastal areas considered to be favorable environments for *V. cholerae* growth; although, other inland sites (Cul-de-Sac Plain) suitable for *V. cholerae* proliferation were also examined.

## Materials and Methods

**Study period**

To search for toxigenic *V. cholerae* present in the Haitian environment, the study was performed during the warmest period of the rainy season (Figure 1). Aquatic samples were collected between July 3 and July 10, 2012, a period characterized by high surface water temperatures. During this period, approximately 168 suspected cholera cases were reported per day in the departments of West (including the Port-au-Prince metropolitan area) and Artibonite (Figure 1).

**Evolution of the daily suspected cholera cases in the departments of West (including Port-au-Prince conurbation) and Artibonite, daily accumulated rainfall in the area and the daily mean temperature in Port-au-Prince in 2012. Time point of the sampling period (July 3 to 10 2012).**
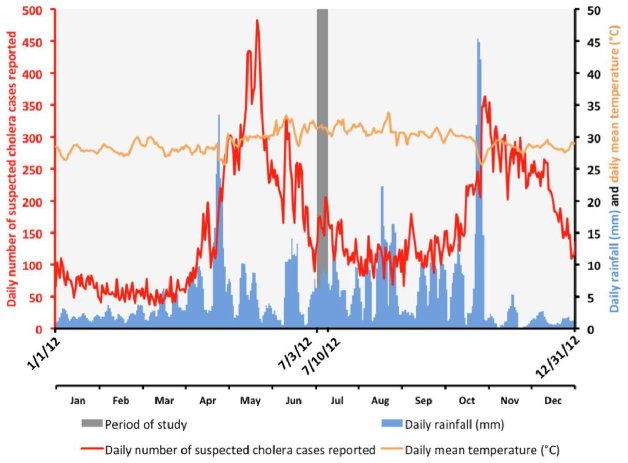
Accumulated rainfall data were obtained from satellite estimates (TMPA-RT 3B42RT derived) averaged on the position 18.25N-19.75N / 74.25W-71.75W and available at http://disc2.nascom.nasa.gov/Giovanni/tovas/realtime.3B42RT_daily.2.shtml (accessed April 29, 2013). Mean daily temperatures observed at Port-au-Prince airport were obtained from the following: http://gis.ncdc.noaa.gov/map/viewer/#app=cdo&cfg=cdo&theme=temp&layers=1&node=gis (accessed April 29, 2013).

**Sampling sites**

Thirty-six stations were sampled from 4 distinct areas (Figure 2, Figure 3). The sampling sites were selected based on field observations, focusing on the nature of the water bodies (surface water or waste water), excluding well water and domestic tanks. The urban sampling areas included Port-au-Prince (7 stations) in the West Department as well as Pont-Sondé (1 station), L’Estère (1 station) and Gonaïves (4 stations) in the department of Artibonite.

**Characteristics of the sampling stations:**
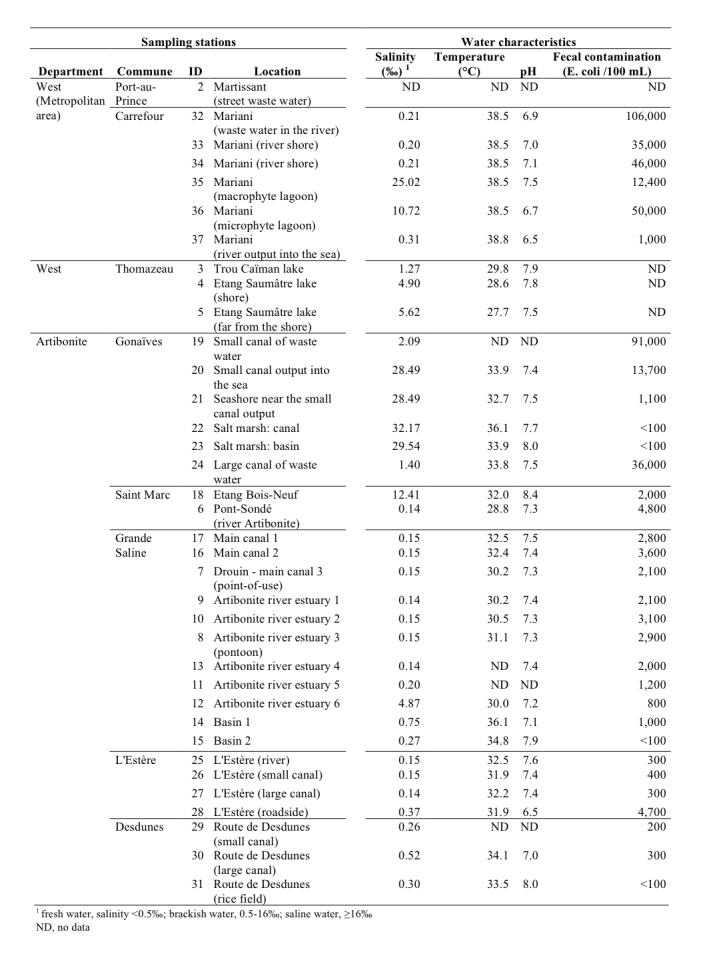
See Figure 3 for localization.

Lakes and ponds unconnected to coastal waters were also tested, as their salinity levels are compatible with *V. cholerae* proliferation. These sites were located at lakes (Trou Caïman and Etang Saumâtre in the Cul-de-Sac plain, 3 stations) and a large pond located southwest of Saint Marc (Etang Bois-Neuf, 1 station). Rural areas of the Artibonite Plain were represented by 17 stations at both the river and diversion canals. Among these 17 stations, 9 were frequently accessed for toilet or laundry activity. The 2 remaining stations were salt marshes located near Gonaïves.

**Localization of the sampling stations in the West and Artibonite departments.**
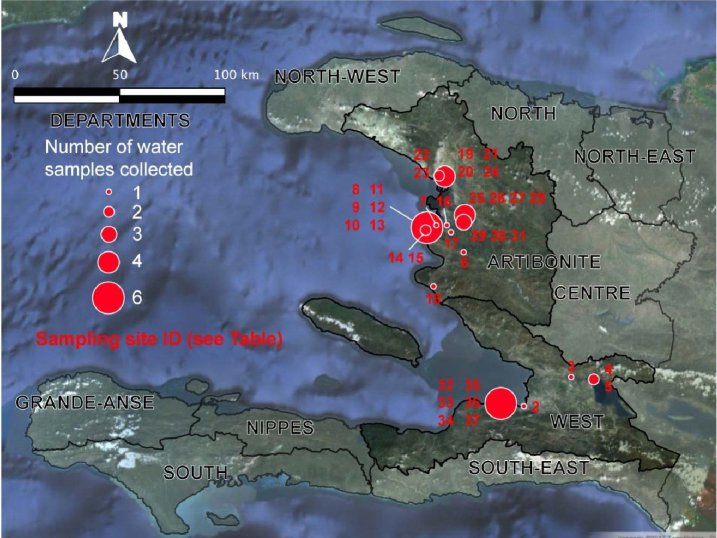
See Figure 2 for the corresponding characteristics.

**Sample collection and processing**

Grab water samples were collected 20 cm below the surface with sterilized narrow-mouth plastic bottles. Sample collection was performed by boat at Etang Saumâtre Lake, Etang Bois-Neuf Pond and the Artibonite estuary. The other samples were collected from the shore with a telescopic pole. Water samples were transported in a cooler (containing frozen packs at the bottom for minimum contact with the bottles) to the Haitian National Laboratory of Public Health (LNSP), and the analysis was performed within 6 to 24 hours of collection.

Surface water temperature and pH levels were measured at the sampling sites using a field pH meter (Hanna HI-98127, Grosseron, Nantes, France); conductivity was assessed at the laboratory with a field conductometer (Hanna HI-99301, Grosseron, Nantes, France). Fecal contamination was determined using Petrifilm™ Select *E. coli* (Département Microbiologie Laboratoires 3M Santé, Cergy, France), which was incubated overnight at 42°C.

**Enrichment and selective cultures**

The analyzed serial volumes for each water sample were selected based on the type of water and the expected abundance of *V. cholerae*. At 6 stations, only a 1-L sample volume was analyzed. At 10 other stations, a 1-L sample volume was analyzed in association with smaller volume samples (100 mL, 10 mL and 1 mL). For the remaining 20 stations, the range of sample volumes analyzed included 100 mL and smaller sample volumes (e.g., 10 mL, 1 mL and sometimes even smaller sample sizes).

Sample volumes of 1 L and 100 mL were filtered (Diaphragm pump N035.3 AN.18 KNF Neuberger, Village-Neuf, France) successively with glass microfiber filters GF/D (grade D, 2.7 µm; Whatman, Maidstone, UK), glass microfiber filters GF/C (grade C, 1.2 µm; Whatman, Maidstone, UK) and 0.45 μm cellulose ester membranes (Millipore, Watford, UK). The filters were sequentially used for the filtration of water samples. The various filter sizes guaranteed the isolation of both fixed-form and free-living bacteria. The filters were placed in 250 mL of sterile Alkaline Saline Peptone Water (ASPW; composition for 1 liter: 10 g peptone, 20 g NaCl and 5 g yeast extract; post-autoclave pH: 8.6 ± 0.2). Sample volumes of 10 mL were incorporated in 100 mL of ASPW, and sample volumes of 1 mL to 0.001 mL were incorporated in 10 mL of ASPW.

The enrichment cultures were incubated from 16 to 24 hours at 41 ± 1°C 12 and subsequently cultured on selective TCBS (Thiosulfate Citrate Bile Sucrose) agar (Difco, provided by Bio-Rad, Marne la Coquette, France) to isolate *V. cholerae* colonies.

The screening procedure was based on phenotypic traits. Up to 20 sucrose-fermenting colonies were transferred with sterile toothpicks onto nutrient agar without NaCl (NA_0 _– Difco, provided by Bio-Rad, Marne la Coquette, France) to test for growth at 37°C and then submitted for an oxidase test (Bactident oxidase strips, Merck, Darmstadt, Germany). All sucrose-fermenting isolates that were able to grow on NA_0_ agar and tested oxidase-positive were considered to be presumptive isolates of *V. cholerae* 13.

**Isolate serotyping**

Presumptive *V. cholerae* isolates were examined to determine whether they were members of the O1 serogroup via slide agglutination using a polyclonal antibody specific for the O1 surface antigen (Bio-Rad, Marne la Coquette, France). A saline solution was used as a control to identify self-agglutinating isolates.

**Molecular identification**

At the LNSP laboratory, DNA extraction of the enrichment cultures was performed automatically after adding 10 µL proteinase K to 200 µL of each enrichment broth using Boom technology (TANBead viral auto kit, Taïwan) with the robot Medipro super pure system-32 (Taïwan). All 107 DNA extracts obtained from 32 sampling sites (stations 6 to 37), both pure and 10-fold dilutions, were assessed via two multiplex PCR assays 14 ^,^ 15. The detection of *V. cholerae* species was performed via PCR targeting a gene encoding an outer membrane protein (*ompW*) 14 (Figure 4). Two different cholera toxin gene (*ctxA*)-specific PCR assays were used to detect the cholera toxin 14 ^,^ 15 (Figure 4). The gene coding for the O1 and O139 surface antigens (*rfb*) was assessed via PCR using O1- and O139-specific primers 15 (Figure 4). The PCR assays were conducted using a G-Storm thermal cycler (Gene Technologies Ltd, Braintree, UK) with the cycling conditions described in Figure 4.

**Primer sequences and multiplex PCR assay conditions:**
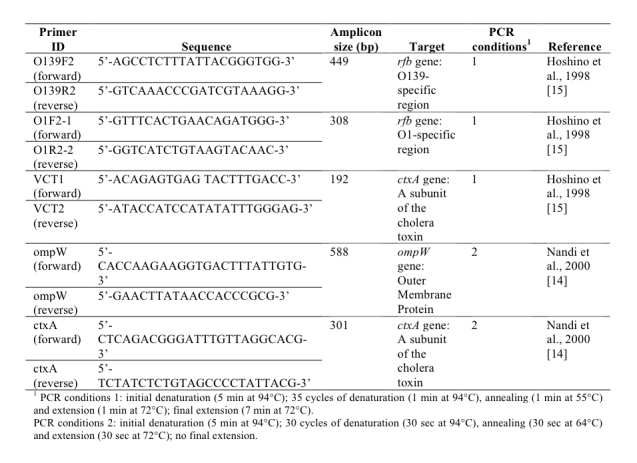

## Results

**Surface water characteristics and fecal contamination**

The disparity in pH levels between the different stations was low (Figure 2), with a mean pH of 7.4 (standard deviation: 0.4; minimum: 6.5 and maximum: 8.4). The Etang Bois-Neuf site displayed the highest water pH level (8.4), which is a pH level known to be favorable for* V. cholerae* growth. The in situ water temperatures ranged between 27.7°C (Etang Saumâtre) and 38.8°C (Mariani) (mean: 33.3°C; standard deviation: 3.3°C). Salinity levels varied greatly between the sampling zones. Therefore, the sampling sites were categorized into 3 groups based on this characteristic: (1) 20 freshwater stations: salinity levels inferior to 0.5‰; (2) 11 brackish water stations (including 3 oligohaline waste waters): salinity levels between 0.5‰ and 16‰ and (3) 5 saline water stations: salinity levels between 16‰ and 40‰.

The levels of fecal contamination varied greatly between samples (Figure 2). Accordingly, the risk of contracting intestinal infections was low in the rice field and salt marshes (less than 100 Colony Forming Units (CFU) per 100 mL), high at the Artibonite plain stations located a pronounced distance from housing (101 to 1000 CFU per 100 mL) and very high near housing settlements (more than 1000 CFU per 100 mL). In waste waters, fecal contamination levels ranged from 10,000 to 100,000 CFU per 100 mL.

**Absence of toxigenic *Vibrio cholerae* O1**

From a total of 141 enrichment cultures derived from the water samples collected at the 36 sampling sites, 411 presumed isolates of *V. cholerae* were isolated. The distribution of the isolates by sampling site is provided in Figure 5. Nine sampling sites failed to yield any* V. cholerae* isolates. Five of these sites (sites 20, 21, 22 and 23 at Gonaïves and site 35 at Port-au-Prince) were saline waters. The other 4 stations (sites 8, 11, 12 and 13), which were fresh waters, were located at the mouth of Artibonite estuary.

**Results of Vibrio cholerae cultures, identifications and PCR assays:**
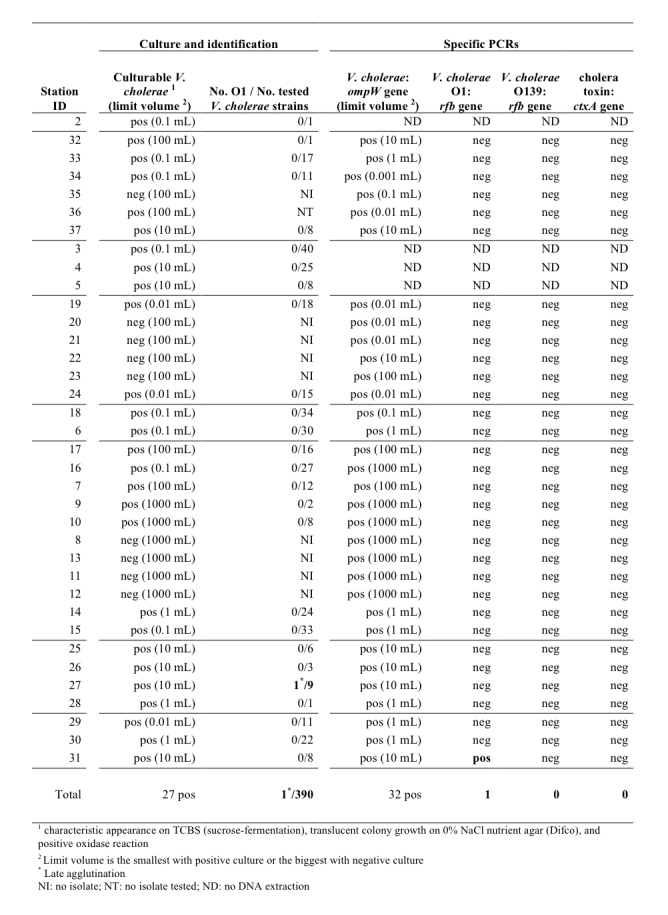

The O1-agglutination test was performed on 390 presumed *V. cholerae* isolates, of which 56 strains were positive for auto-agglutination. Only a single isolate, which was isolated from sampling site 27 (a large canal south of L’Estère), displayed a late positive reaction with polyvalent anti-O1 serum, without auto-agglutination.

The *V. cholerae* PCR assays were found to be more sensitive than the culture assay, as positive results were obtained from all of the 32 tested stations (a total of 77 enrichments out of 107 yielded a positive *V. cholerae­*-specific PCR of the *ompW* gene). However, only 1 sample derived from station 31 in the rice field near Desdunes yielded a positive result for *V. cholerae* O1-specific PCR (*rfb* gene). The *ctxA*-specific PCR was performed on all water enrichments, including from station 27 (where an isolate displayed a late positive reaction with polyvalent anti-O1 serum) and station 31 (positive O1 PCR). However, the cholera toxin was never detected with either pair of *ctxA*-specific primers, even from 100 mL or 1 mL water samples. Therefore, despite the high number of *V. cholerae* isolates obtained, we could not demonstrate the presence of toxigenic *V. cholerae* among all the samples collected.

**Abundance of *Vibrio cholerae* in surface waters and waste waters**

Figure 5 provides the smallest sample volume displaying the presence of *V. cholerae*, or in case of non-detection of *V. cholerae*, the largest volume analyzed for each station. The results of *V. cholerae* detection via culture or PCR are consistent with an abundance ranging from 1 to 10^6^ bacteria per liter depending on the type of water.

## Discussion

The aim of this study was to ascertain whether toxigenic *V. cholerae* could be detected in Haitian aquatic systems at concentrations representing a significant risk to the local population. Our purpose was therefore not to prove the absence of toxigenic *V. cholerae* in the environment, a goal that would require other methods and sampling strategies. Aquatic samples characterized by a wide range of salinity levels were collected during a warm period, at both the time point and locations one would expect to find a high abundance of *V. cholerae* in the environment. Sampling sites were located in Haitian areas profoundly affected by cholera, and most of locales presented medium to high fecal contamination levels, thereby presenting aquatic conditions appropriate for the study of contamination by the epidemic *V. cholerae* clone.

To enhance culturable *V. cholerae* detection, environmental water samples were enriched. Sample enrichment facilitates the detection of *V. cholerae* (including culturable toxigenic *V. cholerae*) regardless of the bacterial form (i.e., free-living bacteria or bacteria attached to phytoplankton, zooplankton and copepods). Various dilutions of the samples were enriched to maximize the chances of isolating and identifying *V. cholerae* clones, and every enrichment culture was analyzed.

Moreover, as it has been reported that cholera bacterium are also found in a viable but non-culturable state in the environment 16 ^,^ 17, we also performed several PCR assays on each enrichment culture to detect toxigenic* V. cholerae* bacteria that may remain non-culturable. The application of both bacterial culture and PCR techniques on enrichment samples has been proposed by other groups 18 ^,^ 19. This two-sided approach has proven to be effective as every station presented positive results by either one method or the other with respect to the presence of *V. cholerae*. However, the two approaches may yield conflicting results for technical reasons; a gene associated with a specific phenotype may be detected via PCR, while it may remain unexpressed or absent in isolated colonies. The inverse situation is also possible, as we analyzed diverse aliquots. It is probably for these reasons that we observed two discrepancies between selective culture and PCR regarding the detection of *V. cholerae* O1 with samples 27 and 31. Finally, to improve our chances of detecting toxigenic *V. cholerae*, we used two distinct PCR assays targeting the *ctxA* gene and systematically tested both pure and 10-fold dilutions of the DNA extracts.

Nevertheless, despite all the precautions that were taken, we found no evidence of the presence of toxigenic *V. cholerae* O1 in any of the samples collected. The absence of the *ctxA* gene not only highlights the absence of *V. cholerae* cholera toxin-positive bacterium, but it also suggests the absence of phages carrying the gene in water samples. These findings seem to contrast with those described by Hill et al. (2011) who isolated 2 culturable toxigenic *V. cholerae* O1 strains from two 30-L water samples among 14 samples 19. However, these two studies were not carried out in the same context. Hill et al. searched for toxigenic *V. cholerae* during the first epidemic wave in October-November 2010, when attack rates of cholera exceeded 2,000 new cases per day, with an epicenter around the Artibonite coast 3 ^,^ 7. At that time, many more infected individuals were likely to contaminate the environment with toxigenic *V. cholerae* via open-air defecation compared with the July 2012 period.

In contrast with the absence of toxigenic *V. cholerae* O1, numerous non-toxigenic non-O1 *V. cholerae* isolates were isolated in our study, even in very small sample volumes. Our results indicate that non-toxigenic *V. cholerae* are well established in freshwater and brackish Haitian aquatic environments. This is not surprising, as it has been demonstrated that the *V. cholerae* species can be isolated from many aquatic ecosystems throughout the world, including cholera-free areas, whether in freshwater 20 ^,^ 21, brackish water 22 ^,^ 23 ^,^ 24, seawater 25 ^,^ 26 ^,^ 27 or even waste water 28.

Our study shows that in July 2012 the bacterial levels of the imported toxigenic clone were far below the levels required for direct transmission to local human populations, despite the massive biomass disseminated in 2010-2011 by more than half a million cholera patients in a country where open-air defecation 29 and the washing of clothes in rivers are widely practiced. The true level of exposure required to contract cholera is difficult to precisely assess. In a study performed in rural Bangladesh, Spira et al. (1980) have shown that people infected during the course of the study were unlikely to have ingested more than 10^5^ viable organisms per day 30, whereas a study by Cash et al. (1974) has established 10^4^ as the minimum inoculum required to provoke diarrhea in healthy volunteers with neutralized gastric acid 31. In our study, the lack of toxigenic *V. cholerae* O1 detection using PCR assays on enrichment cultures was well established for 31 stations with 100-mL or 1-L sample volumes. This strongly suggests that all water samples analyzed contained less than 10 toxigenic *V. cholerae* bacteria per liter, a level 1000-fold below the dose that has been shown by Cash et al. to provoke diarrhea in healthy adults with neutralized gastric acid 31. Notably, because well water and domestic tanks were excluded from our sampling design, our findings do not preclude the possibility of higher levels of toxigenic *V. cholerae* O1 in peri-domestic water bodies following recent contamination by infected individuals.

Non-toxigenic *V. cholerae*, such as those identified in the Haitian aquatic environment in this study, may provoke gastroenteritis or sporadic cholera-like diarrhea in humans; however, these strains have never been implicated in large-scale cholera epidemics 32. Only *V. cholerae* serogroup O1, both ‘classical’ and ‘El Tor’ biotypes, and the derivative serogroup O139 are known to cause cholera epidemics 32. The relationship between all cholera isolates implicated in the seventh pandemic has recently been elucidated by Mutreja et al. (2011) in a study based on whole-genome sequencing of 154 *V. cholerae* strains collected from all over the world 33. By analyzing high-resolution markers (genome-wide single nucleotide polymorphisms), they showed that all strains isolated from various outbreaks during the seventh pandemic have a single common ancestor that emerged during the 1950s. Over time, the clones diversified. Most strains disappeared within a few years, while the remaining strains gave way to new pandemic waves spread by human activity. As we demonstrate that the imported epidemic *V. cholerae* strain has failed to settle in high levels in the aquatic environment of Haiti, our results are in total accordance with the Mutreja et al. findings. If the epidemic strains disseminated by humans could gain a foothold in the environment for an extended duration and eventually proliferate to levels compatible with epidemic reactivation via environment-to-human contamination, phylogenetic assessment of the 154 *V. cholerae* isolates analyzed by Mutreja et al. would not have revealed the diversification and extinction phases that characterized the distinct pandemic waves since the emergence of the seventh pandemic 33.

According to Faruque and Mekalanos, the precursors of the pandemic clones probably displayed traits that are lacking in environmentally adapted *V. cholerae*, regardless of the serogroup 32. As specified by these authors, the evolution of environmental strains into typical pathogenic strains would require more widespread gene transfer events than that shown to occur with known phages 32. Inversely, the transmission of a toxigenic *V. cholerae* O1 strain could be dependent on the amplification-via-disease lifestyle, and the inability of the bacteria to re-establish in the environment might be due to the requirement of human host-dependent replication and transmission. Importantly, a recent study has found that the Haitian *V. cholerae* strain has failed to acquire any genes via horizontal gene transfer from the population of non-toxigenic *V. cholerae* bacteria residing in the local aquatic environment, thereby suggesting that environmental strains have probably played no role in the evolution of the outbreak strain 34.

In fact, our findings suggest that despite its massive dissemination, the toxigenic strain imported into Haiti may no longer be present in the environment at levels required for transmission to humans.

In conclusion, these findings provide hope that cholera could be eliminated from Hispaniola with the recovery of the last patient. Such an objective seems all the more realistic as the elimination of epidemic-causing *V. cholerae* strains has already been observed in Latin American countries such as Peru and Mexico 35. Mexican coasts present many aquatic environments conducive to *V. cholerae* proliferation, and many rural populations still suffer from limited access to potable water and suitable health care 29. As over 43,000 cases were reported in Mexico with a higher incidence in coastal states from 1991 to 1996, Mexico was predicted to become a cholera-endemic region 36. However, this pessimistic prediction failed to materialize, and the annual cholera incidence throughout the entire country dwindled down to 5 cases by the year 2000 37 and 1 case the following year 38. Strikingly, the disease has not been observed in this supposedly endemic country since 2001, and has obviously been extinguished in Mexico. Based on the current observations, the same outcome also seems plausible for Haiti, where cholera outbreaks during the rainy season appear to reemerge from persistent transmission foci insufficiently tackled during the dry season 8.

## Correspondence

**Renaud PIARROUX**

APHM –Hôpital de la Timone, Laboratoire de Parasitologie-Mycologie

264, rue Saint Pierre

13385 MARSEILLE cedex 05

FRANCE

E-mail: renaud.piarroux@ap-hm.fr
